# A Model System for Feralizing Laboratory Mice in Large Farmyard-Like Pens

**DOI:** 10.3389/fmicb.2020.615661

**Published:** 2021-01-11

**Authors:** Henriette Arnesen, Linn Emilie Knutsen, Bente Wabakken Hognestad, Grethe Marie Johansen, Mats Bemark, Oliver Pabst, Anne Kristine Storset, Preben Boysen

**Affiliations:** ^1^Faculty of Veterinary Medicine, Norwegian University of Life Sciences, Oslo, Norway; ^2^Faculty of Chemistry, Biotechnology and Food Science, Norwegian University of Life Sciences, Aas, Norway; ^3^Department of Microbiology and Immunology, Institute of Biomedicine, Sahlgrenska Academy, University of Gothenburg, Gothenburg, Sweden; ^4^Region Västra Götaland, Sahlgrenska University Hospital, Department of Clinical Immunology and Transfusion Medicine, Gothenburg, Sweden; ^5^Institute of Molecular Medicine, RWTH Aachen University, Aachen, Germany

**Keywords:** animal model, mice, feral mice, feralized mice, trained immunity, immune experience, gut micobiota, naturalistic environment

## Abstract

Laboratory mice are typically housed under extremely clean laboratory conditions, far removed from the natural lifestyle of a free-living mouse. There is a risk that this isolation from real-life conditions may lead to poor translatability and misinterpretation of results. We and others have shown that feral mice as well as laboratory mice exposed to naturalistic environments harbor a more diverse gut microbiota and display an activated immunological phenotype compared to hygienic laboratory mice. We here describe a naturalistic indoors housing system for mice, representing a farmyard-type habitat typical for house mice. Large open pens were installed with soil and domestic animal feces, creating a highly diverse microbial environment and providing space and complexity allowing for natural behavior. Laboratory C57BL/6 mice were co-housed in this system together with wild-caught feral mice, included as a source of murine microbionts. We found that mice feralized in this manner displayed a gut microbiota structure similar to their feral cohabitants, such as higher relative content of Firmicutes and enrichment of Proteobacteria. Furthermore, the immunophenotype of feralized mice approached that of feral mice, with elevated levels of memory T-cells and late-stage NK cells compared to laboratory-housed control mice, indicating antigenic experience and immune training. The dietary elements presented in the mouse pens could only moderately explain changes in microbial colonization, and none of the immunological changes. In conclusion, this system enables various types of studies using genetically controlled mice on the background of adaptation to a high diversity microbial environment and a lifestyle natural for the species.

## Introduction

The common habitat for the house mouse *(Mus musculus)* is on the ground, typically close to larger animals like humans and their livestock, and the genetic basis for all research mice evolved in such environments (Boursot et al., 1993). Colonization by a host-specific microbiota is necessary to develop essential parts of the mucosal immune system in mice (Cebra, 1999; Chung et al., 2012), and expression of effector- as well as tolerance-associated immune genes are upregulated following microbial colonization (El Aidy et al., 2012). Nevertheless, mice are usually studied under strictly hygienic laboratory conditions. Hence, concerns have been raised whether hygienically raised laboratory (lab) mice will reach a level of immune maturation that fully recapitulates the immune response in a mammal (Tao and Reese, 2017). Large variations in microbiota and cellular composition of the gut mucosa have been observed between animal facilities, accompanied by different immune phenotypes and experimental performance (Ivanov et al., 2009; Kriegel et al., 2011; Jakobsson et al., 2015; Rausch et al., 2016; Franklin and Ericsson, 2017). Thus, an artificial between-lab variability may have replaced natural variability in the course of comprehensible efforts to standardize the world’s most used animal model.

Theories have postulated that a modernized lifestyle has led to a loss of proximity to a diverse range of microbes and parasites, thus removing balancing factors in the immune homeostasis, which may explain an increase of inflammatory diseases and cancer (Hunter, 2020). A major current research field addresses how colonizing microbes, including bacteria, parasites and even viruses, may affect the immune system to generate a lasting and general protection from various diseases. Beyond specific immunity, recent evidence shows how innate immune cells may undergo long-lasting reprogramming following microbial challenges, sometimes referred to as trained immunity (Oh et al., 2014; Honda and Littman, 2016; Netea et al., 2020). Adaptive immune cells may also be primed in a similar manner (Muraille and Goriely, 2017). The concept of immune training has been associated with enhancement of immune responses to vaccines and infections as well as to anti-inflammatory actions (Quinn et al., 2019). The outcome of immune training for a particular disease may thus point in either direction and needs to be explored empirically in organisms exposed to diverse microbial cues.

This background gives a rationale to develop animal models reflecting more realistic ecological contexts (Flies and Wild Comparative Immunology Consortium, 2020). In contrast to the widespread use of hygienically raised inbred mice, studies investigating the microbiota and immunity of mice under more naturalistic conditions have only recently emerged. We and others have demonstrated that feral (wild-caught) mice had an immunological steady state different from lab mice (Devalapalli et al., 2006; Abolins et al., 2011, 2017, 2018), as well as a thicker mucus layer in the gut (Jakobsson et al., 2015). In an effort to decipher the impact of environment, one study found profound changes in the immune system of inbred mice following co-housing with “dirty” pet store mice, approaching phenotypes found in feral mouse as well as adult humans (Beura et al., 2016). In another, pre-infection of inbred mice with selected common mouse pathogens resulted in stronger vaccine responses (Reese et al., 2016). Furthermore, by transplanting feral mouse feces (Rosshart et al., 2017) or by transferring microbiota vertically from feral surrogate mothers (Rosshart et al., 2019), “wildling” lab mice were shown to develop a trained immune system and increased protection against disease. The latter study demonstrated the translational gain by using naturalized mice, as wildling mice behaved immunologically human-like in two clinical settings where conventional lab mice had failed to predict the response. Another study showed that the provision of soil heaps in mice cages modified the gut microbiota and repressed Th2-driven inflammation, in support of the “hygiene hypothesis” (Ottman et al., 2019). However, in all the studies mentioned above, lab mice remained in conventional cages, with limitations of space and behavioral opportunities relative to a wild house mouse lifestyle. A recently described model where mice were kept in large outdoor enclosures, showed altered microbiota, a shift toward Th1-type immunity and an increased susceptibility to helminth infection (Leung et al., 2018; Lin et al., 2020; Yeung et al., 2020). While offering a habitat clearly representing wild conditions, this setup allows limited surveillance of the animals and may prove inaccessible for most researchers.

We present a naturalistic environment housing system for mice consisting of large indoor enclosures (pens) containing farmyard-like elements such as fecal content from farm animals, soil and plant materials, with spatial living conditions reflecting a natural habitat. In a set of experiments, C57BL/6 mice were feralized under these conditions in the presence of feral house mice, serving as a natural source of mouse microbes, including pathogens and parasites. We show that feralization lead to a significant conversion of the gut microbiota composition, and to immunological parameters associated with antigenic experience and immune training.

## Materials and Methods

### Animals and Experimental Design

**FIGURE 1:**
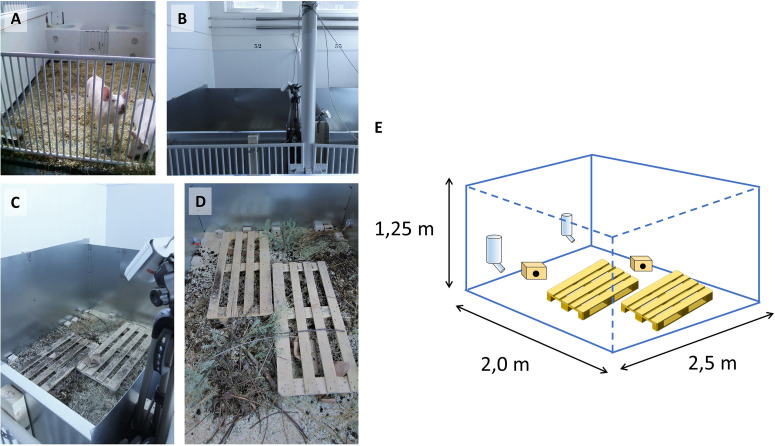
Construction of mouse pens. Original pig pens in the large animal clinic at NMBU shown in **(A)** were modified with steel sheets **(B,C)** and equipped with surveillance cameras **(B,C)**. **(D)** Contents of mouse pens as outlined in section “Materials and Methods.” **(E)** Schematic representation of pen construction, showing pallets, wooden houses and drinking bottles (pallet graphics from https://publicdomainvectors.org/).

A mouse pen housing system was designed at The Norwegian University of Life Sciences (NMBU) by escape-proofing pig pens with sheets of galvanized steel, each pen measuring 2.0 × 2.5 × 1.25 m (WxDxH) on concrete floor (Figures 1A–E and Supplementary Video S1). Pens were enriched with wood shavings, organic garden soil, compost, twigs, hay and fecal content from pigs, cows and horses. Oat and carrot sprouts were planted occasionally to provide fresh plants as would be encountered in a farmyard. Wooden pallets were used as stepping platforms for personnel to avoid disturbing the habitats or crushing animals, also contributing to environmental complexity and shelter. Standard nippled drinking bottles provided water. Small wooden boxes were provided for nesting and sheltering. In Experiment (Exp.) 1, surveillance cameras with infrared sensors were used for continuous monitoring.

Following purchase, C57BL/6N (B6) specific pathogen-free (SPF) mice (Charles River/Scanbur, Norway) were acclimatized for 1 week in individually ventilated cages (IVCs) under SPF conditions at NMBU. Feral house mice were captured in domestic animal farms in south-eastern Norway by overnight deployment of Ugglan Special No1 live traps (Grahnab, Gnosjö, Sweden), equipped with wood shavings, fresh fruit and peanut butter as bait, as previously described (Boysen et al., 2011). Representatives of these catches were subtyped as *Mus musculus* ssp. *musculus*, with a minor contribution of ssp. *domesticus* as reported previously (Knutsen et al., 2019). The ages of feral mice could not be determined, but only visibly adult individuals were included. Mice were individually marked using ear punch or microchip injected subcutaneously (Nonatec Lutronic, Rodange, Luxembourg). Feral and B6 mice were released simultaneously into pens.

Experiments and housing design were approved by the National Animal Research Authority in Norway (FOTS 4788, 6801, and 8080). Feral mice capture was approved by The Norwegian Directorate for Nature Management (2012/693 and 2014/7215).

**FIGURE 2:**
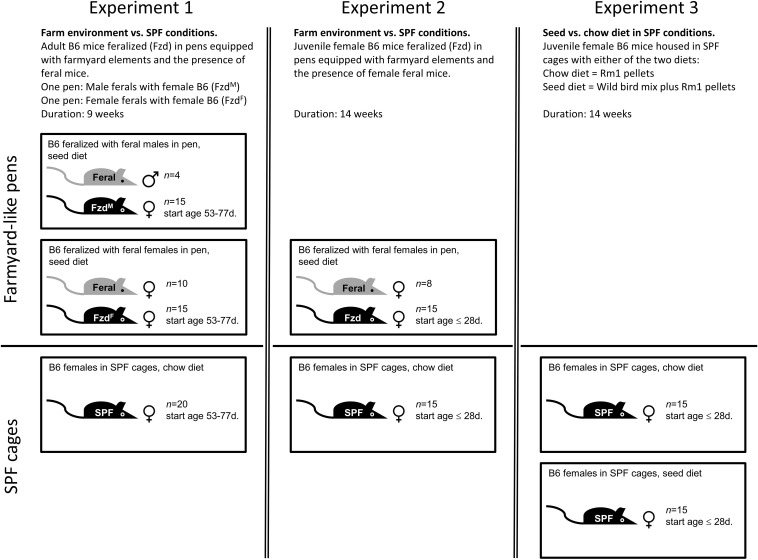
Graphical representation of the experimental design of the three experiments described in this study, as detailed in section “Materials and Methods.”

In Exp. 1 (Figure 2), female B6 mice aged 53–77 days were feralized by housing in pens together with feral mice for 9 weeks, divided into two subgroups: In one pen, 15 female B6 mice were co-housed with 10 female feral mice (Fzd^F^; feralized with feral females). In a second pen, 15 female B6 mice were co-housed with 4 male feral mice (Fzd^M^; feralized with feral males). Fzd^M^ mice produced several litters of hybrid offspring excluded from the study. To provide a diet reflecting food sources in a natural setting, we provided an unprocessed wild bird seed mix consisting of sunflower seeds (25%), sorghum (25%), oat (25%), and wheat (25%) (Wild bird mix, Plantasjen, Köping, Sweden), mixed with standard “chow” pellets (Rm1, Special Diet Services, United Kingdom/Scanbur, Norway) *ad lib* on the ground (see Supplementary Table S1 for nutrient composition.) In addition, pen mice had access to a variation of plant material, including dried hay, spruce twigs collected outdoors, and occasional fresh lettuce, carrots and fruits. 20 female B6 mice of the same cohort were housed in cages under SPF conditions as controls, receiving standard chow diet only, to maintain typical lab conditions.

In Exp. 2 (Figure 2), 15 female B6 mice aged 28 days were feralized in mouse pens with 8 female adult feral mice for 14 weeks (Fzd), while 15 B6 females were kept in cages as SPF controls. Feeding regimen as described above. As the feralized mice were fed a natural diet in the previous experiments, we designed Exp. 3 (Figure 2) to assess the effects of the major dietary sources of the previous two experiments, carried out in IVCs under conventional lab conditions. 30 female B6 mice (source, age and gender as in Exp. 2) were housed for 14 weeks in cages of 5 mice per cage. The animals were randomized into two groups receiving either chow or a combinatory diet of chow and seed mix (the latter hereafter referred to as seed group for simplicity).

The mice were exposed to human caretakers in the pens on a daily basis, but direct handling was minimized, and mice were not re-captured until termination of the experiment. Only mice that were clinically healthy condition at termination were included in the studies. All mice were euthanized by neck dislocation, followed by immediate exsanguination by cardiac puncture, weighing and measuring, and dissection of sample tissues.

### Isolation of Cells and Serum

Cells harvested from tissues using a GentleMACS dissociator and mouse Spleen Dissociation Kit (Miltenyi Biotech, Bergisch Gladbach, Germany) according to the manufacturer’s instructions. Splenic suspensions were briefly treated with NH_4_Cl solution to lyse erythrocytes. Single-cell suspensions were prepared using a 70 μm cell strainer (BD Biosciences) and concentrations standardized after counting using a Countess automated cell counter (Thermo Fisher Scientific). Serum was isolated from blood following centrifugation of clotted whole blood at 3,000 g for 5 min.

### Microbial Community Analyses

For microbial community analyses, fecal pellets were flash frozen in liquid N2 after collection and stored at −80°C. DNA extraction, library preparation and 16S rDNA 454 pyrosequencing were conducted as described previously (Lindner et al., 2015). Briefly, DNA was isolated and purified with QIAamp DNA Stool Mini Kit (Qiagen) according to manufacturer’s manual. Libraries we generated with a primer set covering the V1–V3 regions of the 16s rRNA gene (8F/541R). 16S rRNA gene amplicons were purified by gel electrophoresis followed by gel extraction (QIAquick Gel Extraction kit, Qiagen). Amplicons were prepared with the GS FLX Titanium SV emPCR kit (Lib-A) for 454 pyrosequencing on the Genome Sequencer FLX system (Roche) according to manufacturer’s instructions. In Exp. 2 and 3, feces was collected from all individuals at baseline (t0) and termination following 14 weeks of feralization (t1).

Raw reads were processed using the Integrated Microbial Next Generation Sequencing (IMNGS) pipeline (Lagkouvardos et al., 2016) based on the UPARSE approach. Briefly, sequences were demultiplexed, trimmed to the first base with a quality score > 3 and paired. Sequences with > 1000 nucleotides and assembled reads with expected error of > 3 were excluded from the analyses (Exp. 2, USEARCH 8.0; Exp. 3, USEARCH 8.1) (Edgar, 2010). Remaining reads were trimmed by 10 nucleotides at forward and reverse end. The presence of chimeras was tested with UCHIME (Edgar et al., 2011). Operational taxonomic units (OTUs) were clustered at 97% sequence similarity (Edgar, 2010) (Exp. 2, USEARCH 8.0; Exp. 3, USEARCH 8.1), and only those with a relative abundance of > 0.50% (Exp. 2) or > 0.25% (Exp. 3) in at least one sample were kept. Taxonomies were assigned at 80% confidence level with the RDP classifier (Wang et al., 2007) (version 2.11, training set 15). Sequences were aligned with MUSCLE (Edgar, 2004) and trees were generated with Fasttree (Price et al., 2010). In Exp. 2 the analyzed dataset included 1,207,683 quality- and chimera-checked sequences ranging from 6,527 to 48,172 per sample, representing a total of 338 OTUs. One individual in the Fzd group was excluded from analyses due to abnormally high sequence depth (152,009). In Exp. 3 the analyzed dataset included 3,481,304 quality- and chimera-checked sequences ranging from 39,504 to 131,663 per sample, representing a total of 220 OTUs. Sequencing files from Exp. 2 and Exp. 3 are deposited to the Sequence Read Archive and are available under the accession number PRJNA668303.

### Flow Cytometry and *in vitro* T-Cell Stimulation

Immunophenotyping was carried out by incubating single-cell suspensions in phosphate-buffered saline (PBS) with 0.5% bovine serum albumin and 10 mM NaN3 on ice. After FcR-blocking with anti-CD32/16 antibody (eBioscience), cells were stained with Live/Dead Fixable Yellow Dead Cell Stain Kit (Thermo Fisher Scientific) followed by incubation with combinations of monoclonal antibodies as listed in Supplementary Table S2. For intracytoplasmatic staining, cells were treated with Intracellular Fixation and Permeabilization Buffer Set, or with Foxp3/Transcription Factor Staining Buffer Set for intranuclear antigens (both eBioscience), according to the manufacturer’s instructions. Fluorescence levels were measured using a Gallios 3-laser flow cytometer and analyzed using Kaluza 1.2 software (Beckman Coulter). Cells were gated as shown in Supplementary Figure S1, using single cell staining, omission of antibodies and matched isotypes as controls. For stimulation assays, splenocytes were cultured at concentration of 2 × 10^6^ cells/ml together with Dynabeads Mouse T-Activator CD3/CD28 (Thermo Fisher Scientific) at a 1:1 ratio, in RPMI (Gibco) l-glutamine supplemented with 60 μg/ml penicillin, 100 μg/ml streptomycin, 1 mM sodium pyruvate, 50 μM 2-mercaptoethanol, non-essential amino acids (all Gibco/Invitrogen), 10% fetal calf serum (PAA) and 30 U/ml recombinant murine (rm)IL-2 (eBioscience) for 48 h. Brefeldin A (10 μg/ml; Sigma) was added 4 h before harvesting, followed by immunophenotyping.

### Multiplex Assays

Cytokines were measured in serum using the following multiplex assays: Bio-Plex Pro Mouse Cytokine 8-plex panel (#M60000007A) supplemented with IL-6 and IL-17A singleplex, Bio-Plex Pro^TM^ TGF-β 3-plex Assay (#171W4001M) (Bio-Rad), or ProcartaPlex Th1/Th2/Th9/Th17/Th22/Treg Cytokine 17-Plex Mouse Panel (EPX170-26087-901) (Thermo Fisher Scientific). Antibody subclasses were measured using ProcartaPlex Mouse Antibody Isotyping 7-plex panel (EPX070-20815-901). In all cases the analyses were carried out following the manufacturers’ instructions, using a Luminex 200 reader and BioPlex Manager 6.0 software (BioRad). Analysis was performed on fluorescence index (FI) values minus background, while figures show concentrations calculated from standard curve. Analytes with more than 40% data points below limit of detection (Antweiler, 2015) were excluded from statistical evaluation.

### Statistical Analyses

Microbial profiles and composition were analyzed in the R programming environment (R version 4.0.2) (R_Core_Team, 2020) using Rhea (Lagkouvardos et al., 2017)^1^. To account for differences in sequence depth, OTU tables were first normalized by dividing each sample’s reads to their total reads, then multiplication by the total reads of the smallest sample. *Beta*-diversity was calculated based on generalized UniFrac distances (Chen et al., 2012) and the significance of separation between groups was tested by permutational multivariate analysis of variance (PERMANOVA). *Alpha*-diversity was assessed based on species richness and Shannon effective diversity as described in detail in Rhea. Only taxa with a prevalence of ≥ 30% (proportion of samples positive for the given taxa) in one given group, and relative abundance ≥ 0.25% were considered for statistical testing. For analyses of differences in relative abundance between > 2 groups (Exp. 2), Kruskal-Wallis Rank Sum test was performed. A significant Kruskal-Wallis test (*p* < 0.05) was followed by pairwise Wilcoxon Rank Sum tests. *P*-values were corrected for multiple comparisons according to the Benjamini-Hochberg method, and adjusted *p*-values are reported. For comparisons of two groups (Exp. 3), Wilcoxon Rank Sum tests were performed directly. For analyses of differences in prevalence between groups, Fisher’s exact tests were performed. Over-time analyses within groups were performed using paired Wilcoxon Signed Rank Sum tests.

**FIGURE 3:**
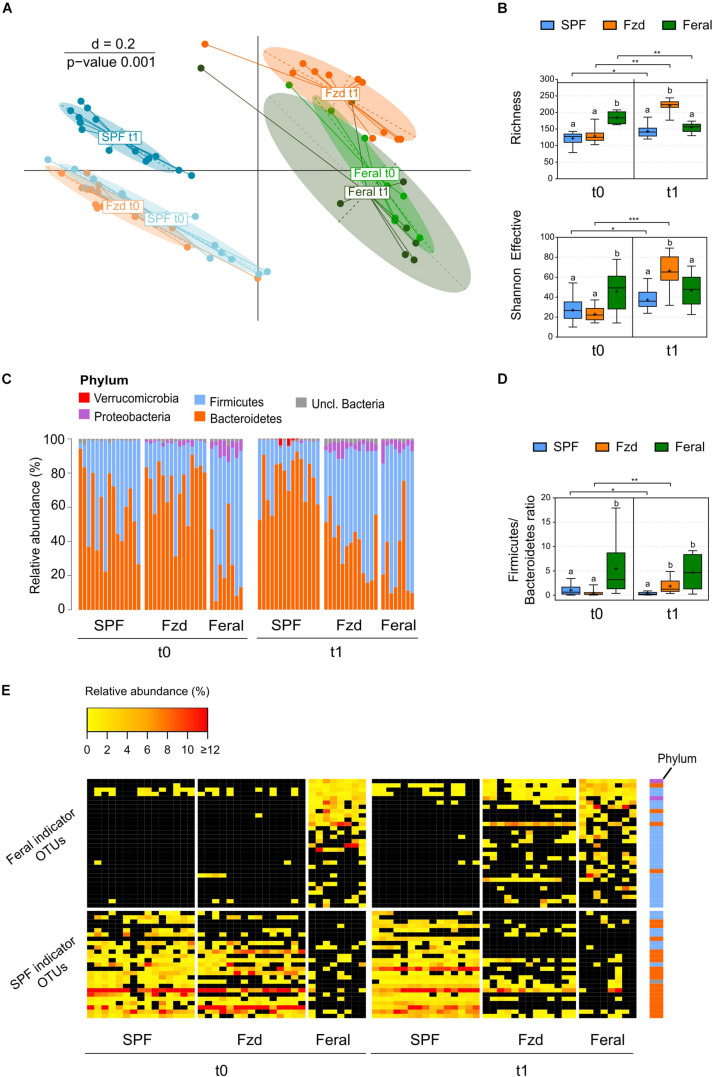
Feralization lead to a gut microbiota diversity and composition converging with feral mice. Presented data is from Exp. 2. **(A)** Multi-dimensional scaling (MDS) plot of microbiota profiles for feral, feralized (Fzd) and SPF mice at baseline (t0) and termination (t1). Similarities between profiles were computed using generalized Unifrac distances. The significance of separation between groups was tested by PERMANOVA. d = dissimilarity scale. **(B)** Richness (observed OTUs) and Shannon effective diversity index. Box plots show median (line), mean (+), IQR (box) and minimum to maximum (whiskers). Asterisks designate over-time differences determined by Wilcoxon Signed-Rank Sum test. Differences between groups at each timepoint were determined by Kruskal-Wallis and Mann-Whitney *U*-tests. The Benjamini-Hochberg method was used to correct for multiple testing. Levels not sharing the same letter were significantly different at α = 0.05. ^∗^*p* ≤ 0.05, ^∗∗^*p* ≤ 0.01, ^∗∗∗^*p* ≤ 0.001. **(C)** Taxonomic binning at the rank of phylum, presented as relative abundance for each individual, with groups and timepoints indicated. **(D)** Firmicutes/Bacteroidetes ratio presented as in **(B)**. **(E)** Heatmap of relative abundance of Feral- and SPF-associated OTUs identified by indicator species analysis. Phyla of which the OTUs belong to are designated with colored squares specified in **(C)**. Relative abundances of the OTUs < 0.25% were set to NA (black). All plots: *n* = 8 (feral) or *n* = 13–15 (other groups).

In order to identify patterns of differentially abundant and prevalent OTUs in Feral and SPF mice, we conducted an indicator species analysis implemented by the *indicspecies* package (De Cáceres and Legendre, 2009) in R. The significance of the associations was determined by permutation tests followed by Benjamini-Hochberg correction of resulting *p*-values. To identify highly indicative OTUs, we included only OTUs that occurred in ≥ 70% of the mice in either the Feral or SPF group at each timepoint. For Exp. 3, an indicator species analysis was conducted in the same manner as described for Exp. 2, to identify OTUs indicative of Chow-fed or Seed-fed animals independent of timepoint. For all groups at both timepoints, the relative abundances of the identified indicator-OTUs were plotted with the *heatmap.2* function from the *gplots* package (Warnes et al., 2020) in R. The closest species related to the indicator-OTU sequences were identified with EzBioCloud (Yoon et al., 2017). See Supplementary Table S3 for a complete list of indicator-OTUs presented in Figure 3E and Supplementary Figure S4E.

Immunological data was analyzed using log (Ln)-transformed values. Comparisons between groups were performed using the statistical applications JMP v.14 (SAS Institute Inc.) or Prism v.7 (GraphPad Software, Inc.), applying Student’s *t*-test for two groups, and Tukey-Kramer’s multiple comparison test for > 2 groups, at alpha level 0.05, unless otherwise stated. In figures with letter indications, levels not sharing the same letter were significantly different. Multivariate analyses were performed using the principal component analysis (PCA) on Correlations, and hierarchical clustering using Ward’s minimum variance method in JMP on the variables listed in Supplementary Table S4, excluding one Fzd mouse with an incomplete data set.

## Results

### Lab Mice Adapted Well to a Farmyard Habitat in the Presence of Feral Mice

Throughout Exp. 1 we closely monitored how animals performed through direct inspection and using surveillance or handheld cameras (Supplementary Figure S2). Feral and B6 mice were released simultaneously into the pens to avoid biased territorializing (Supplementary Video S2). The mice dug holes in the soil that appeared preferential for nesting rather than using wooden houses provided for this purpose (Supplementary Video S3). Feral and B6 mice mingled well, in both the male-female and the female-female setups. Feral mice generally reacted to human presence by hiding, re-emerged within few minutes and approached people (Supplementary Video S4), whilst the B6 mice were generally less shy. Feral mice quickly adapted to drinking from water bottles. However, four feral individuals were found dead with no visible signs of injuries and lack of water being a possible cause. No B6 mice died, showed visible bruises or signs of disease, except one slow-moving Fzd^M^ female that was excluded from the study. Fzd^M^ females mated with feral males and produced litters that were cared for in a shared dirt-hole nursing colony. However, since past or present pregnancy might confound the readouts, we chose to carry out subsequent experiments in an all-female setting. In Exp. 2 the observed behavior was similar to Exp. 1, and all introduced mice were recaptured in healthy condition.

### Feralized Mice Acquired Mouse Pathogens and a Feral-Like Gut Microbiota

Serum samples from four individuals of each mouse group in Exp. 1 were screened for antibodies against a range of pathogens. Feral mice carried antibodies for Minute virus of mice (MVM), Mouse parvovirus (MPV), Mouse Cytomegalovirus (MCMV) and, in one case, *Pasteurella pneumotropica* (*Pp*) (Supplementary Table S5A). Fzd^M^ mostly seroconverted to mimic the feral mice, while only a single Fzd^F^ mouse tested positive for one pathogen (*Pp*). SPF controls were negative for all tested agents. A gross parasitological examination of intestines with fecal content revealed the presence worms or eggs in feral and Fzd^M^ mice, but to a less extent in Fzd^F^ mice while negative in SPF controls (Supplementary Table S5B).

The terminal gut microbiota in stool samples from Fzd^F^ mice in Exp. 1 has been reported previous (Lindner et al., 2015). Briefly, the microbiota profile of the feralized mice approached that of feral mice, including a higher relative abundance of Firmicutes and Proteobacteria, while SPF mice stood out with a separate profile. Data from Exp. 2 largely mirrored the findings of Exp. 1. At baseline, *beta*-diversity analysis demonstrated a distinct clustering of baseline gut microbiota of the B6 mice separate from feral mice (Figure 3A), and *alpha*-diversity measures showed a significantly higher number of observed OTUs (richness) in feral mice compared to the Fzd and SPF groups (both *p* ≤ 0.001) (Figure 3B). At the rank of phylum, a significantly higher relative abundance of Firmicutes and lower relative abundance of Bacteroidetes was detected in feral mice compared to Fzd (both *p* ≤ 0.001) and SPF (*p* = 0.035 and *p* = 0.005, respectively), as reflected in a higher Firmicutes/Bacteroidetes ratio (Figures 3C,D). Moreover, Proteobacteria abundance above cutoffs were detected in all feral mice and the majority of feralized mice, but only in one SPF individual (Figure 3C). In feral mice, the Proteobacteria was mainly accounted for by two OTUs with closest sequence similarity to *Helicobacter* species (*Helicobacter ganmani*, 99.6% similarity; *Helicobater typhlonius*, 100% similarity), while in Fzd the Proteobacteria was mainly accounted for by one OTU with the closest sequence similarity to *Kiloniella laminariae* (86.3% similarity).

A clear shift in the microbiota profile was seen following feralization, in which the Fzd mice approached a Feral-like profile (Figure 3A). Feralization led to a dramatic increase in both richness and Shannon effective (*p* = 0.002 and *p* ≤ 0.001, respectively), indicating an elevated number of species representing a higher level of phylogenetic diversity (Figure 3B). An increase in relative abundance of Firmicutes and decrease in relative abundance of Bacteroidetes (both *p* = 0.001) was observed following feralization, reflected in an increased Firmicutes/Bacteroidetes ratio (*p* = 0.005) (Figures 3C,D). The shift following feralization was further supported by analysis of the terminal gut microbiota, in which the Fzd and feral mice demonstrated significantly higher *alpha*-diversity measures and Firmicutes/Bacteroidetes ratios, and increased relative abundances of Proteobacteria compared to the SPF mice (Figures 3C,D). Moreover, we conducted an Indicator Species Analysis to identify OTUs that were most indicative for Feral and SPF mice based on the probability of occurrence and abundance in these groups independent of timepoint. This algorithm was first developed by Dufrene and Legendre (1997) and has been employed previously to track persistence of OTUs in mice following environmental changes (Seedorf et al., 2014) and fecal microbiota transfer from wild to laboratory mice (Rosshart et al., 2017). Generally, the OTUs associated with Feral mice belonged to the Firmicutes phylum, while the SPF-associated OTUs were members of Bacteroidetes, mirroring the detected phylum-level differences (Figure 3E). Two OTUs with closest sequence similarities to *Helicobacter* species (*Helicobacter ganmani, 99.6%; Helicobater typhlonius, 100%*) were identified as Feral-associated OTUs (Figure 3E and Supplementary Table S3A). By plotting the abundances of the indicator OTUs for all samples, we were able to track the Feral-associated and SPF-associated OTUs in the Fzd group over time. Prior to feralization, the Fzd and SPF groups showed overlapping patterns, with high abundance of SPF-associated and generally low abundance of Feral-associated OTUs. Following feralization, a substantial proportion of Feral-associated OTUs was detected, while only a very few SPF-associated OTUs undetected in Feral mice remained in the Fzd group at endpoint (Figure 3E).

Taken together, feralization led to a substantial change in gut microbiota structure, approaching the profile and composition seen in feral mice. Seropositivity to viral pathogens was detected in all feral mice, and in female feralized mice co-housed with feral males, but not in those co-housed with feral females.

### Feralization Lead to Immunophenotypes Consistent With Antigenic Experience and Immune Training

**FIGURE 4:**
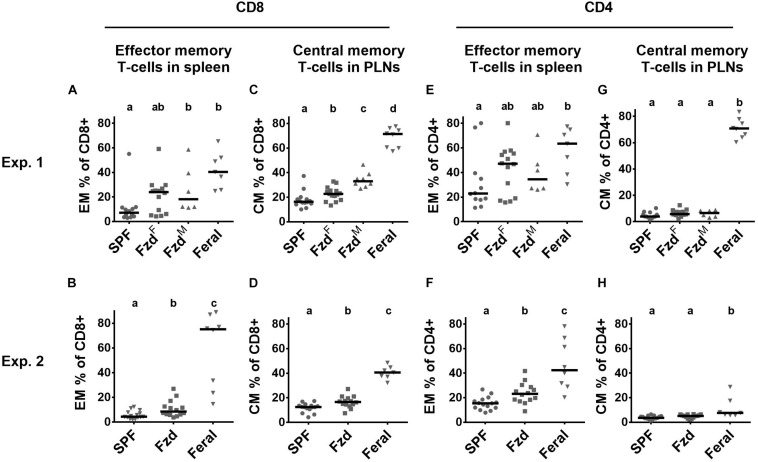
Memory T-cell subsets were accumulated in feralized mice. Cellular phenotypes were measured by flow cytometry, gated as indicated in Supplementary Figure S1. Data from Exp. 1 and 2 is shown respectively as follows: **(A,B)** CD8^+^ EM T-cells in spleen; **(C,D)** CD8^+^ CM T-cells in PLNs; **(E,F)** CD4^+^ EM T-cells in spleen; **(G,H)** CD4^+^ CM T-cells in PLNs. PLNs, peripheral lymph nodes; Exp., Experiment; EM, Effector memory; CM, Central memory; SPF, Specific pathogen free; Fzd^F^ or Fzd, Female B6 feralized with female feral mice; Fzd^M^, Female B6 feralized with male feral mice. Levels not sharing the same letter were significantly different at α = 0.05.

**FIGURE 5:**
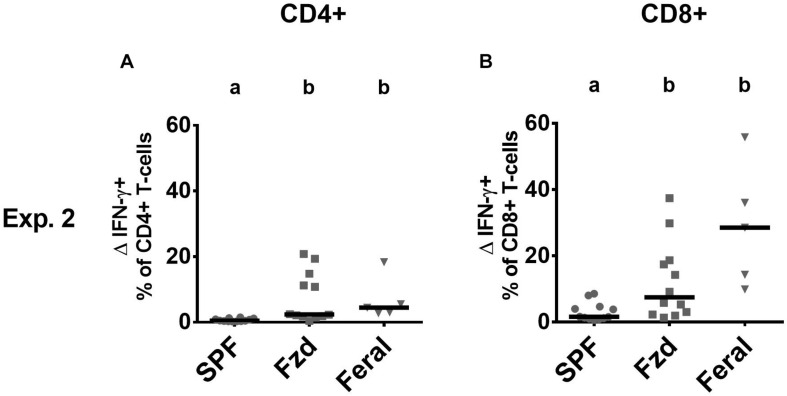
T-cells in feralized mice responded to in vitro stimulation with increased interferon gamma (IFN-γ) production. Splenocytes were incubated for 48 h with bead-coupled anti-CD3/CD28 antibodies in the presence of IL-2 and measured for intracellular IFN-γ production by flow cytometry. ΔIFN-γ^+^ (IFN-γ^+^ NK cells in stimulated cultures—ditto in medium control cultures) is shown as per cent of CD4^+^ T-cells **(A)** or of CD8^+^ T-cells **(B)**. All data from Exp. 2. Abbreviations and statistics as in Figure 4.

Cellular phenotypes were measured according to gating strategies shown in Supplementary Figure S1. In both Exp. 2 and Exp. 3, the number of T-cells and CD4^+^ and CD8^+^ subsets were similar in feralized and SPF mice in SPL as well as peripheral lymph nodes (PLNs) (not shown). Memory T-cells, defined as CD44^+^CD62L^+^ central memory (CM) cells, or CD44^+^CD62L^–^ effector memory (EM) cells were measured in the spleen and PLNs, respectively, according to the most common compartments for these subsets (Wherry et al., 2003; Stockinger et al., 2006). Feralized mice showed increased levels of CD8^+^ T cells with an EM phenotype in the spleen (Figures 4A,B) as well as CM cells in the PLNs (Figures 4C,D). A tendency for increased proportions of EM CD4^+^ cells was seen in the spleen of feralized mice (Figures 4E,F), but not for CM CD4^+^ cells in the PLNs (Figures 4G,H). Feral mice consistently had more cells displaying an EM or CM phenotype within the CD8 as well as the CD4 subsets (Figures 4A–H). To assess if T-cells of feralized mice had changed their potency as effector cells, we cultured splenocytes with anti-CD3/CD28 coupled beads for 48 h in the presence of IL-2 in Exp. 2. The frequency of interferon-gamma positive CD8^+^ and to a lesser extent CD4^+^ T-cell populations was higher in feralized mice compared to SPF mice (Figures 5A,B).

**FIGURE 6:**
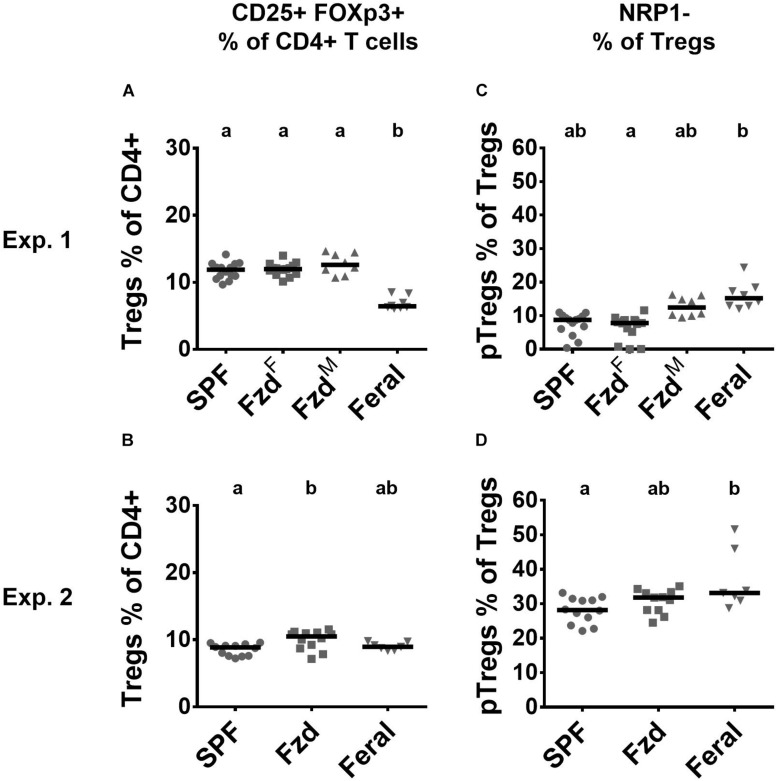
Regulatory T-cell numbers and phenotypes in peripheral lymph nodes were largely unaffected by feralization. Data from Exp.1 and 2 is shown respectively as follows: **(A,B)** Relative numbers of Tregs as per cent of CD4^+^ T-cells; **(C,D)** Proportion of Tregs defined as pTregs according to lack of NRP1 expression. Tregs, Regulatory T-cells; pTregs, peripheral Tregs; NRP1, Neuropilin-1; otherwise abbreviations and statistics as in Figure 4.

Regulatory T-cells (Tregs) (CD3^+^CD4^+^CD25^+^Foxp3^+^) were measured in PLNs. In Exp. 1, feralized mice had similar number of Tregs as SPF mice, while feral mice had a lower proportion (Figure 6A). In Exp. 2, slightly elevated Treg numbers were seen in feralized but not in feral mice (Figure 6B). We furthermore, assessed neuropilin-1 (NRP-1) dim or negative cells, associated with peripherally induced regulatory T-cells (pTregs), especially induced by gastrointestinal exposure (Bilate and Lafaille, 2012). In both Exp. 1 and 2, the proportion of pTregs was slightly elevated in the feral mice, but insignificantly so in feralized mice (Figures 6C,D).

**FIGURE 7:**
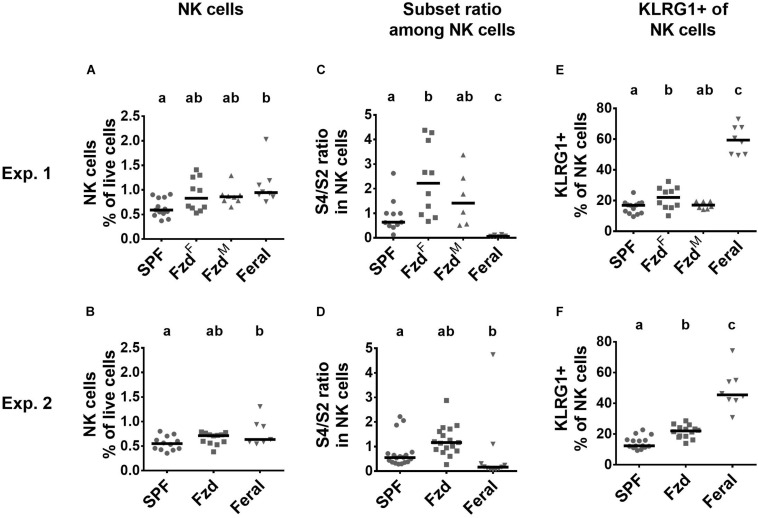
Natural killer (NK) cells in feralized mice showed signs of maturation. Maturational subsets were defined as shown in Supplementary Figure S1, with subset ratio calculated based on CD27^+^CD11b^–^ (S4)/CD27^–^CD11b^+^ (S2). Data from Exp.1 and 2 is shown respectively as follows: **(A,B)** Relative numbers of NK cells as per cent of live cells; **(C,D)** Ratio of S1/S2 subsets of NK cells within NK cells; **(E,F)** Proportion of NK cells expressing KLRG1. Abbreviations and statistics as in Figure 4.

NK cells numbers were elevated in PLNs but not spleens of feral mice (Figures 7A,B and Supplementary Figures S3A,B), as observed previously (Boysen et al., 2011). In feralized mice NK cells tended to increase, albeit not statistically significant, in the PLNs (Figures 7A,B), while no differences were observed in the spleen (Supplementary Figures S3A,B). Murine NK cells can be phenotypically divided into maturation stages as early (S1) CD27^–^CD11b^–^, mid (S2) CD27^+^CD11b^–^, late (S3) CD27^+^CD11b^+^, and fully mature (S4) CD27^–^CD11b^+^ stages (Chiossone et al., 2009; Abolins et al., 2017), most cells normally found within the S2–S4 categories. We found that feral mice had a decreased S4/S2 ratio in both PLNs and in spleen, as seen previously (Boysen et al., 2011). In contrast, increased S4/S2 ratio was detected in feralized mice, most evident in the PLNs (Figures 7C,D and Supplementary Figures S3C,D). KLRG1 expression was elevated in NK cells in feral mice in PLN (Figures 7E,F) and partly in spleen (Supplementary Figures S3E,F), confirming previous observations (Boysen et al., 2011). To a lesser extent, feralized mice also had elevated KLRG1 in PLNs (Figures 7E,F), a tendency also evident in spleen (Supplementary Figures S3E,F).

**FIGURE 8:**
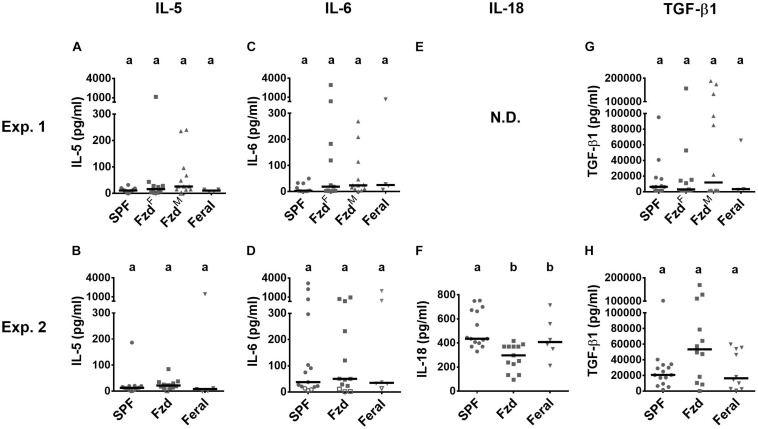
Cytokines in serum were minimally affected by feralization. Serum samples were measured by multiplex assays. Data from Exp.1 and 2 is shown respectively as follows: **(A,B)** IL-5; **(C,D)** IL-6; **(E,F)** IL-18; **(G,H)** TGF-β. Open symbols indicate measurements below accurate quantification. N.D., No data. Otherwise abbreviations and statistics as in Figure 4.

Most tested serum cytokines were low and not significantly altered between groups (Figure 8). However, IL-18 was lower in the feralized and feral mice (Figure 8F). A tendency of increased TGF-β1 in feralized mice was noted but with high variability and not statistically confirmed (Figures 8G,H). Some additional cytokines were either not significantly altered or fell below the lower limit of detection (Supplementary Table S6).

**FIGURE 9:**
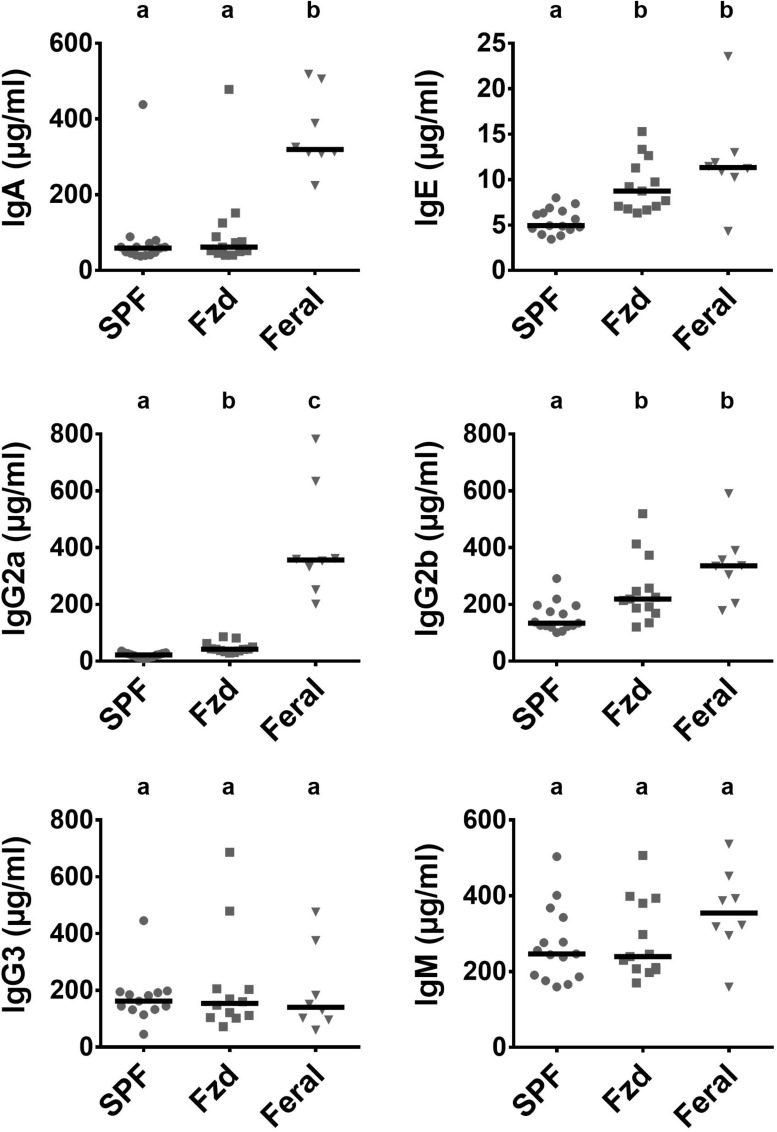
Antibody isotypes IgE, IgG2a, and IgG2b were elevated in feralized mice. Presented data is from Exp. 2. Isotypes were measured in serum by multiplex immmunoassay. Abbreviations and statistics as in Figure 4.

Increased serum levels of IgE and IgG1 have previously been reported in feral mice (Devalapalli et al., 2006; Abolins et al., 2011), and in Exp. 2 we measured immunoglobulin subclasses using a multiplex assay, demonstrating that feral mice had increased serum IgA, IgE, Ig2a, Ig2b, and IgM (Figure 9). Feralized mice showed a tendency of increased IgE and IgG2b, while the remaining subclasses fell within the same range as SPF controls. IgG1 was not detected above background levels (not shown).

**FIGURE 10:**
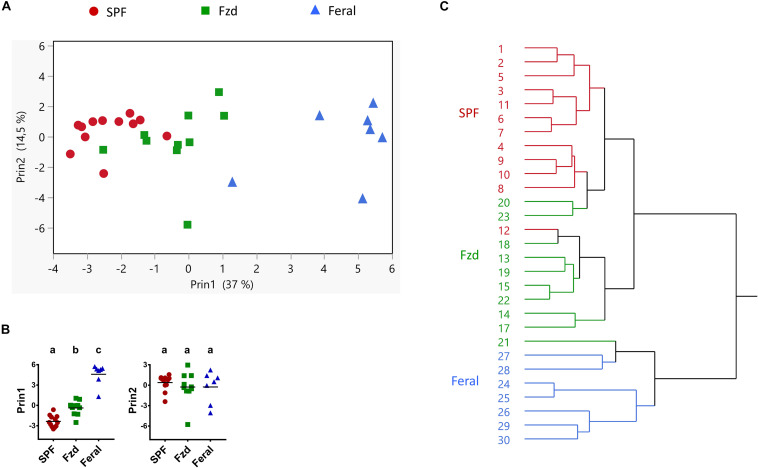
Multivariate analyses of immune parameters showed that feralized mice clustered in the direction of feral mice. Presented data is from Exp. 2. **(A)** Principal coordinate analysis of Ln transformed values of variables listed in Supplementary Table S3. **(B)** Post-PCA analysis of the two first PCA variables (Prin1 and Prin2) using Tukey-Kramer’s multiple comparison for all groups. Different letters designate statistical significance with alpha level 0.05. **(C)** Hierarchical cluster analysis, numbers refer to individual mouse IDs. Fzd = Female B6 feralized with female feral mice.

The data from Exp. 2 had a completeness that allowed multivariate analysis of immune parameters, in order to explore any co-variation undetected when assessing single parameters. A PCA analysis revealed that feralized mice grouped separately from SPF controls, in the direction of feral mice (Figure 10A), significantly different between all groups in first principal component (Prin1) but not Prin2 (Figure 10B). Likewise, a cluster analysis grouped mice from each study group separately, except a minor overlap between SPF and feralized mice (Figure 10C).

### Diet May Explain Shifts in Gut Microbiota, but Was Not Found to Drive Immunological Changes

To assess the contributions of differing diets between groups in Exp. 1 and 2 to gut microbiota and immunophenotypes, Exp. 3 was designed to incorporate the two diets in a SPF lab cage setting. Microbial profiling of feces showed a shift in microbiota composition and increased *alpha*-diversity measures on both diets, although more prominently in mice fed the seed diet compared to regular chow (Supplementary Figures S4A,B). At the rank of phylum, no significant changes were detected in the chow-fed animals. For the seed-fed animals, we detected a significantly decreased relative abundance of Bacteroidetes (*p* = 0.013) and increased relative abundance of Firmicutes (*p* = 0,011), reflected in increased Firmicutes/Bacteroidetes ratio over-time (*p* = 0.048) (Supplementary Figures S4C,D). Similar to Exp. 2, the over-time changes were supported by analyses of the terminal gut microbiota, in which seed-fed mice demonstrated significantly higher *alpha*-diversity measures and Firmicutes/Bacteroidetes ratio compared to the chow-fed mice. However, in contrast to the findings from Exp. 2, relative abundances of Proteobacteria were unchanged following the different diets. In the analysis of data from Exp. 3 we also conducted an indicator species analysis to identify OTUs that were most indicative for the Chow-fed and Seed-fed mice independent of timepoint. Relatively few indicator-OTUs were identified in this analysis, indicating only small differences between Chow- and Seed-fed mice at the OTU-level (Supplementary Figure S4E and Supplementary Table S3B). At baseline, prior to administration of different diets, the two groups showed overlapping patterns (Supplementary Figure S4E). From baseline to endpoint, Seed-fed animals showed an enrichment of several OTUs belonging to the phylum Firmicutes, mirroring the findings at phylum-level. Whilst we found changes in gut microbiota following the two diets, immunophenotyping showed no diet-induced difference in CD44^+^ cells in the CD8^+^ T-cell compartment. In the CD4^+^ T-cell subset, a minor increase of CD44^+^ cells was observed in seed-fed mice (Supplementary Figure S5A). In *ex vivo* CD3/CD28 stimulated splenocytes, intracellular IFN-g production was similar in the two diet groups in both CD8^+^ or CD4^+^ T-cells (Supplementary Figure S5D). Moreover, we observed no significant effect of diet on Treg levels or pTreg proportions, NK cells or NK cells subsets (Supplementary Figures S5B,D).

Taken together, the findings from Exp. 3 indicated that a diet similar to that given to feralized mice in Exp. 1 and 2 caused a significant shift in gut microbiota structure, yet provided no evidence for any shift in immunological parameters assignable to the diet.

## Discussion

A spacious and naturalistically enriched environment meets an increasing demand to improve housing conditions to refine the experimental output from mouse models (Balcombe, 2010). Large indoor enclosures have previously been used to study house mouse behavior (Gray et al., 2000; Jensen et al., 2003, 2005; Weissbrod et al., 2013), but to our knowledge, no reports describe the microbiological and immunological phenotypes of mice reared in such enclosures enriched as a naturalistic environment. The tremendous adaptability of the house mouse implies that no single habitat is universally “natural.” Nevertheless, house mice are predominantly found in or near human dwellings, farm buildings, food stores and waste areas, and its dispersal largely follows human movements (Pocock et al., 2005). The house mouse often forms high-density territories as small as a few square meters (Selander, 1970). To set up a well-defined naturalistic scenario we constructed large pens containing essential farmyard elements through domestic animal fecal material, soil and plants, and with wild-caught mice present as microbial donors. The aim of the current report was to observe the performance of laboratory mice housed in this model system and to observe their resulting fecal microbiota and key elements of their systemic immunity phenotype.

Feralization led to a significant shift in gut microbiota composition and increased *alpha*-diversity measures following feralization, supportive of previous reports of microbially exposed mice (Ottman et al., 2019; Liddicoat et al., 2020). We observed an enrichment of Proteobacteria in feral and feralized mice, in agreement with findings in “wildling” B6 mice born to feral mothers (Rosshart et al., 2019) as well as B6 mice co-housed with pet store mice (Huggins et al., 2019). Two OTUs associated with feral mice microbiota showed the closest similarity to *Helicobacter* species and were enriched in feralized mice. In a recent paper, *Helicobacter* species have been suggested to trigger colonic T cell responses in a context-dependent manner (Chai et al., 2017). Moreover, the higher Firmicutes/Bacteroidetes ratios in feralized and feral mice corresponds to a previous report of feral mice (Kreisinger et al., 2014), but contrasts the lower relative abundance of Firmicutes seen in fecal samples from wildlings (Rosshart et al., 2019), from B6 mice receiving fecal transfer from wild mice (Rosshart et al., 2017), as well as from soil-exposed mice (Ottman et al., 2019). However, care should be taken when interpreting between studies, as geographical factors have been shown to drive the microbiota composition to a larger extent than genotypes, including between *Mus musculus* subspecies (Linnenbrink et al., 2013; Kreisinger et al., 2014). Notably, feral mice maintained a similar microbiota before and after pen housing in Exp. 2. Their prior microbial environment and diet is unknown, but they were caught at farms distant from the sources used for enrichment, and these findings could either indicate that the conditions we created reflected their feral situation, or that their colonized microbiota was more resilient to change compared to the SPF-derived laboratory mice.

The seed-based diet offered in the naturalistic environment contained higher amounts of fiber and fat compared to the standard chow diet, both of which are groups of dietary components demonstrated to alter gut microbiota composition and influence immunity in a wide range of previous studies (Daniel et al., 2014; Trompette et al., 2014; Desai et al., 2016; Xiao et al., 2017; Las Heras et al., 2019). We therefore set up a third experiment to assess this impact in an otherwise hygienic context. Seed-fed mice had increased *alpha*-diversity and Firmicutes/Bacteroidetes ratio, suggesting a partial explanation for changes seen in the feralized mice. We did not observe differences in the investigated immune parameters following the two diets, suggesting the alterations of immunophenotypes were driven by other stimuli, or other components of the microbiota, than those conferred by diet.

A multivariate analysis showed that the combined systemic immune phenotype of feralized mice clustered distinctly from SPF mice in the direction of feral mice, albeit still closer to SPFs. For cellular measurements we concentrated on NK and T-cell phenotypes associated with maturation and memory. Feralized and feral mice had increased numbers of memory CD8+ T cells, in line with report of long-lasting expansion following *in vivo* challenge (Vezys et al., 2009). Similar upregulation of memory T cells has been reported in feral and pet-store mice, in lab mice co-housed with pet-store mice and in rewilded mice (Beura et al., 2016; Abolins et al., 2017; Lin et al., 2020). Following *ex vivo* stimulation, we found that T-cells in feralized and feral mice responded more potently with IFN-gamma production compared to lab mice, similar to previous reports in feral and rewilded mice (Abolins et al., 2017; Lin et al., 2020).

We found little effect of feralization on the total Treg cell numbers, while pTregs, defined as NRP-1^–^ Tregs (Bilate and Lafaille, 2012), showed a slightly increasing tendency, not significant in feralized but significant in feral mice. A previous study in feral mice found marginal increase in splenic Tregs (Abolins et al., 2017), while two other studies of microbially diversified lab mice found no alteration in Treg cell numbers (Frossard et al., 2017; Rosshart et al., 2019). These findings suggest that Tregs in systemic organs do not respond substantially to these types of environmental triggers.

NK cells may be activated by pathogens or primed by proinflammatory cytokines, transforming the cells into a more mature state, in many cases persisting as memory-like or trained NK cells (Nabekura and Lanier, 2016; Netea et al., 2020). In mice, trained NK cells have been found to display a mature KLRG1^+^ and CD27^–^CD11b^+^ phenotype (Nabekura and Lanier, 2016). We reproducibly found more CD27^–^CD11b^+^ and KLRG1^+^ NK cells in feralized B6 mice. Notably, as reported previously (Boysen et al., 2011; Abolins et al., 2017), feral mice had a contrasting overweight of CD27^+^CD11b^–^ NK cells, yet with a high KLRG1 expression. Feral and feralized mice underwent the same microbial pressure for 2–3 months, suggesting that the CD27/CD11b discrepancy may be genetic rather than environmental. However, this aberrance is unlikely due to genetic differences amongst subspecies, as while the present mice were *M. m. musculus*-dominated, the same NK cell phenotype have been noted in feral *M. m. domesticus* mice (Abolins et al., 2017), the latter constituting the major genetic background for the B6 mouse (Yang et al., 2011). Regardless of cause, the finding emphasizes the importance of assessing genetically controlled individuals in this type of studies.

Low levels of serum cytokines were detected and these were apparently not sensitive to environmental changes, as also seen in wildlings (Rosshart et al., 2019). The observed elevation of IgE in feral and feralized mice compare with findings from other studies (Devalapalli et al., 2006; Abolins et al., 2011, 2017) and are possibly caused by the presence of parasites as were detected in Exp. 1. Besides parasites, seroconversion for pathobionts were especially evident in the Fzd^M^ females, which made frequent intimate contacts with feral males during mating. In the all-female setup, a feral-type feral microbiota was obtained, yet the serological results indicated that they acquired less of a pathogenic burden. These findings may suggest that relatively strong and/or frequent transmission pressure of pathobionts is needed for a mouse to reach a feral-like immunophenotype.

The scope of the presented studies was to achieve a full-scale naturalistic environment, rather than to explore underlying mechanisms, which would require multiple investigations or a substantial reduction of the very elements that create diversity. Depending on the scope, future studies may add or exclude elements, such as considering the necessity of mouse-specific microbionts obtained through the inclusion of feral mice. While the basis for environmental diversity will inevitably vary between geographical sites (Linnenbrink et al., 2013; Kreisinger et al., 2014), so does the microbiota in highly isolated conventional facilities (Rausch et al., 2016). The strength of genetically controlled model animals remains, and studies on the background of mice feralized in this manner can complement studies in conventional SPF and germ-free lab mice, as demonstrated by us in two reports (Lindner et al., 2015; Monin et al., 2020). By ensuring that environmental materials derive from the same sources throughout the experiment, and preferably between experiments, well-controlled and reproducible experiments can be achieved. A “dirty” environment as described here must in most cases be established separate from clean mouse houses, in our hands successfully achieved in an experimental large animal facility, and later built as a separate satellite unit.

## Conclusion

In conclusion, we have demonstrated how laboratory mice can be feralized in large pens containing feral cohabitant mice, recapitulating a natural mouse habitat. Feralized mice reproducibly carried an altered fecal microbiota and immunophenotype in systemic immune tissues. This model system represents a refinement opportunity for various purposes, such as assessing the performance of infections, drugs or vaccines on the background of “real-world” adapted animals.

## Data Availability Statement

The original contributions presented in the study are publicly available. This data can be found in NCBI, under accession number PRJNA668303.

## Ethics Statement

The animal study was reviewed and approved by National Animal Research Authority, Norwegian Food Safety Authority.

## Author Contributions

PB and AKS designed the research. BWH and PB conducted animal Exp. 1. LEK and PB conducted animal Exp. 2 and 3. BWH, LEK, GMJ, MB, OP, and PB performed the laboratory procedures. HA and PB analyzed the data and wrote the manuscript. All authors contributed to the article and approved the submitted version.

## Conflict of Interest

The authors declare that the research was conducted in the absence of any commercial or financial relationships that could be construed as a potential conflict of interest.

## Footnotes

1 https://github.com/Lagkouvardos/Rhea

## Supplementary Material

Supplementary Figure 1 Flow cytometry gating strategies. **(A)** Single cell, mononuclear cells (MNC) and live cell gates. **(B)** NK cells defined as NKp46+CD3- cells, further defined as maturational stages S1–S4 based on CD27 and CD11b expression, or gated for the expression of KLRG1. **(C)** T-cells gated equivalent to above, gated as CD4^+^ or CD8^+^ and defined as Central Memory (CM; CD62L^+^CD44^+^) or Effector Memory (EM; CD62L^–^CD44^+^). **(D)** Regulatory T-cells, gated on CD4^+^ T-cells equivalent to above, defined as CD25+Foxp3+, and further gated for the expression of Neuropilin-1 (NRP1). **(E)** *In vitro* stimulated T-cells, cultured for 48 h in the presence of CD3/CD28 activator beads and IL-2, gated on T-cells equivalent to above and gated for the expression of interferon gamma (IFNg).

Supplementary Figure 2 Photos from the mouse experiments carried out in the farmyard-like pens. **(A)** Pallet, soil, large animal feces, twigs, straw, feral mice. **(B)** Feral mouse peeking from water bottle. **(C)** Nest with B6 x feral mouse crosses. **(D)** B6 mice checking box (mostly used as toilets; dirt holes were preferred as nests). **(E)** Feral mouse. **(F)** Nighttime grazing on sprouts. **(G)** Offspring playing in box.

Supplementary Figure 3 Natural killer (NK) cell numbers and phenotypes in spleen, equivalent to Figure 7. Abbreviations and statistics as in Figure 4.

Supplementary Figure 4 Gut microbiota diversity and composition of SPF mice fed different diets. Presented data is from Exp. 3. **(A)** Multi-dimensional scaling (MDS) plot of microbiota profiles for seed- and chow-fed animals at baseline (t0) and termination (t1). Similarities between profiles were computed using generalized Unifrac distances. The significance of separation between groups was tested by PERMANOVA. d = dissimilarity scale. **(B)** Richness (observed OTUs) and Shannon effective diversity index. Box plots show median (line), mean (+), IQR (box) and minimum to maximum (whiskers). Asterisks designate over-time differences determined by Wilcoxon Signed-Rank Sum test. Letters designate differences between groups at each timepoint determined by Kruskal-Wallis and Mann-Whitney U tests. The Benjamini-Hochberg method was used to correct for multiple testing. Different letters designate statistical significance with corrected *p* ≤ 0.05. ^∗^*p* ≤ 0.05, ^∗∗^*p* ≤ 0.01, ^∗∗∗^*p* ≤ 0.001. **(C)** Taxonomic binning at the rank of phylum, presented as relative abundance for each individual, with groups and timepoints indicated. **(D)** Firmicutes/Bacteroidetes ratio presented as in **(B)**. **(E)** Heatmap of relative abundances of Chow- and Seed-associated OTUs identified by indicator species analysis. Phyla of which the OTUs belong to are designated with colored squares specified in **(C)**. Relative abundances of the OTUs < 0.25% were set to NA (black). All plots: *n* = 12 (chow) or *n* = 13 (seed).

Supplementary Figure 5 Cellular immunological phenotypes of mice in Exp. 3, corresponding to measurements in main experiments shown in Figures 4–7. Abbreviations and statistics as in Figure 4.

Supplementary Video 1 Mouse pen overview, wooden pallets, soil heaps with fresh domestic animal manure, straw, spruce twigs. / C57BL/6 mice in farm environment.

Supplementary Video 2 Release of feral (wild-caught) mouse. / First encounters between C57BL/6 and feral mice.

Supplementary Video 3 Feral mouse showing agile behavior, use of available space and resources.

Supplementary Video 4 Feral mice showing curiosity, adaptability to B6 mice and people.

## Abbreviations

B6: C57BL/6 inbred mouse strain
CM T-cell: Central memory T-cell
EM T-cell: Effector memory T-cell
Exp. 1/Exp. 2: Experiment 1/Experiment 2
Fzd: Feralized (here: female) B6 mice
Fzd^F^: B6 females feralized in the presence of female feral mice
Fzd^M^: B6 females feralized in the presence of male feral mice
Ig: Immunoglobulin
IL: Interleukin
IVC: Individually ventilated cage
KLRG1: Killer cell lectin-like receptor subfamily G member 1
NRP-1: Neuropilin-1
OTU: Operational taxonomic unit
PCA: Principal component analysis
PLN: Peripheral lymph node
pTreg: Peripherally induced regulatory T-cell
Rm1: Rat and Mouse No.1 diet (^TM^of Special Diet Services)
SPF: Specific pathogen-free
Treg: Regulatory T-cell.
