# Supplementary material for: A Model System for Feralizing Laboratory Mice in Large Farmyard-Like Pens
Source: Front Microbiol. 2021 Jan 11;11:615661. doi: 10.3389/fmicb.2020.615661 (PMC7830425; doi:10.3389/fmicb.2020.615661)
Supplement: Supplementary Figure 1 — Flow cytometry gating strategies. (A) Single cell, mononuclear cells (MNC) and live cell gates. (B) NK cells defined as NKp46+CD3- cells, further defined as maturational stages S1–S4 based on CD27 and CD11b expression, or gated for the expression of KLRG1. (C) T-cells gated equivalent to above, gated as CD4+ or CD8+ and defined as Central Memory (CM; CD62L+CD44+) or Effector Memory (EM; CD62L–CD44+). (D) Regulatory T-cells, gated on CD4+ T-cells equivalent to above, defined as CD25+Foxp3+, and further gated for the expression of Neuropilin-1 (NRP1). (E) In vitro stimulated T-cells, cultured for 48 h in the presence of CD3/CD28 activator beads and IL-2, gated on T-cells equivalent to above and gated for the expression of interferon gamma (IFNg). [file Data_Sheet_1.zip › Supplementary Table S1.pdf]

Supplementary Table S1: Dietary composition

Nutrient	Wild Bird Mix[1]	Rm1[2]
Protein	14,0%	14,4%
Fat	8,5%	2,7%
Fibre	6,5%	4,7%
Ash	1,8%	6,0%
Water	9,8%	10,0%
Remaining (mainly carbohydrates)	59,4%	61,7%

[1] Reported by Plantasjen AB (personal communication)

[2] Special Diet Services (SDS) catalog
