# Supplementary material for: A Model System for Feralizing Laboratory Mice in Large Farmyard-Like Pens
Source: Front Microbiol. 2021 Jan 11;11:615661. doi: 10.3389/fmicb.2020.615661 (PMC7830425; doi:10.3389/fmicb.2020.615661)
Supplement: Supplementary Figure 1 — Flow cytometry gating strategies. (A) Single cell, mononuclear cells (MNC) and live cell gates. (B) NK cells defined as NKp46+CD3- cells, further defined as maturational stages S1–S4 based on CD27 and CD11b expression, or gated for the expression of KLRG1. (C) T-cells gated equivalent to above, gated as CD4+ or CD8+ and defined as Central Memory (CM; CD62L+CD44+) or Effector Memory (EM; CD62L–CD44+). (D) Regulatory T-cells, gated on CD4+ T-cells equivalent to above, defined as CD25+Foxp3+, and further gated for the expression of Neuropilin-1 (NRP1). (E) In vitro stimulated T-cells, cultured for 48 h in the presence of CD3/CD28 activator beads and IL-2, gated on T-cells equivalent to above and gated for the expression of interferon gamma (IFNg). [file Data_Sheet_1.zip › Supplementary Table S4.pdf]

Supplementary Table S4: Parameters included in Figure 10 (PCA)

						Partial contributions in Principal Components Analysis		
**Variable name**	**Description**	**Unit**	**Tissue**	**Method**	**Individual values shown in**	**Prin1**	**Prin2**	**Prin3**
IgA	Immunoglobulin A	µg/ml	Serum	Multiplex assay	Figure 9	6,10589	1,35261	0,00725
IgE	Immunoglobulin E	µg/ml	Serum	Multiplex assay	Figure 9	6,7543	1,25524	0,10601
IgG2a	Immunoglobulin 2a	µg/ml	Serum	Multiplex assay	Figure 9	10,16426	0,0353	0,64977
IgG2b	Immunoglobulin 2b	µg/ml	Serum	Multiplex assay	Figure 9	5,4408	1,74857	0,00298
IgG3	Immunoglobulin G3	µg/ml	Serum	Multiplex assay	Figure 9	0,00109	6,79294	0,01582
IgM	Immunoglobulin M	µg/ml	Serum	Multiplex assay	Figure 9	3,5494	4,04936	0,18368
IL-18	Interleukin-18	pg/ml	Serum	Multiplex assay	Figure 8	0,08069	1,57345	7,89284
IL-6	Interleukin-6	pg/ml	Serum	Multiplex assay	Figure 8	0,25822	7,82576	8,71905
IL-5	Interleukin-5	pg/ml	Serum	Multiplex assay	Figure 8	1,85231	3,15184	17,84695
TGF-b1	Transforming Growth Factor beta 1	pg/ml	Serum	Multiplex assay	Figure 8	0,14981	23,73131	1,49706
TGF-b2	Transforming Growth Factor beta 2	pg/ml	Serum	Multiplex assay	Not shown	0,36945	21,84297	0,70538
TGF-b3	Transforming Growth Factor beta 3	pg/ml	Serum	Multiplex assay	Not shown	0,54113	23,49544	0,51443
NK_PLN	NK cells	% of live mononuclear cells	PLNs	Flow cytometry	Figure 7	2,15801	0,47061	14,94577
KLRG+NK_PLN	KLRG1+	% of NK cells	PLNs	Flow cytometry	Figure 7	8,92832	0,14125	0,07181
S4/S2_NK_Ratio_PLN	Ratio of S4/S2 maturation stages	in NK cells	PLNs	Flow cytometry	Figure 7	4,61427	0,0174	0,60187
NK_SPL	NK cells	% of live mononuclear cells	SPL	Flow cytometry	Supplementary figure S3	2,50179	0,14949	4,07013
KLRG+NK_SPL	KLRG1+	% of NK cells	SPL	Flow cytometry	Supplementary figure S3	7,8618	0,77693	0,40806
S4/S2_NK_Ratio_SPL	Ratio of S4/S2 maturation stages	in NK cells	SPL	Flow cytometry	Supplementary figure S3	0,2075	0,24067	21,94945
CM_of_CD4+_PLN	Central memory type	% of CD4+ T-cells	PLN	Flow cytometry	Figure 4	4,79786	0,05424	8,343
CM_of_CD8+_PLN	Central memory type	% of CD8+ T-cells	PLN	Flow cytometry	Figure 4	9,4957	0,00002	1,72624
EM_of_CD4+_SPL	Effector memory type	% of CD4+ T-cells	SPL	Flow cytometry	Figure 4	8,58756	0,39002	0,33886
EM_of_CD8+_SPL	Effector memory type	% of CD8+ T-cells	SPL	Flow cytometry	Figure 4	9,73512	0,61128	0,08441
Treg_of_CD4+	Regulatory T-cells	% of CD4+ T-cells	PLN	Flow cytometry	Figure 6	0,72392	0,00804	3,88124
NRP1-_of_Treg	NRP-1-	% of Regulatory T-cells	PLN	Flow cytometry	Figure 6	5,12082	0,28525	5,43795
