# Supplementary material for: A Model System for Feralizing Laboratory Mice in Large Farmyard-Like Pens
Source: Front Microbiol. 2021 Jan 11;11:615661. doi: 10.3389/fmicb.2020.615661 (PMC7830425; doi:10.3389/fmicb.2020.615661)
Supplement: Supplementary Figure 1 — Flow cytometry gating strategies. (A) Single cell, mononuclear cells (MNC) and live cell gates. (B) NK cells defined as NKp46+CD3- cells, further defined as maturational stages S1–S4 based on CD27 and CD11b expression, or gated for the expression of KLRG1. (C) T-cells gated equivalent to above, gated as CD4+ or CD8+ and defined as Central Memory (CM; CD62L+CD44+) or Effector Memory (EM; CD62L–CD44+). (D) Regulatory T-cells, gated on CD4+ T-cells equivalent to above, defined as CD25+Foxp3+, and further gated for the expression of Neuropilin-1 (NRP1). (E) In vitro stimulated T-cells, cultured for 48 h in the presence of CD3/CD28 activator beads and IL-2, gated on T-cells equivalent to above and gated for the expression of interferon gamma (IFNg). [file Data_Sheet_1.zip › Supplementary Table S5.pdf]

## Supplementary Table S5

## A. Serology for common pathogens[1]

| | | | Fzd$^M$ | | | | Fzd$^F$ | | | | SPF | | | | Feral males | | Feral females | | F1 pups | |
|---|---|---|---|---|---|---|---|---|---|---|---|---|---|---|---|---|---|---|---|---|
| Pathogenic agent | | *Method* | 609 | 610 | 615 | 614 | 804 | 806 | 808 | 814 | 305 | 306 | 308 | 309 | 011 | 012 | 102 | 106 | 703 | 705 |
| Virus | **MHV** | *IFA* | - | - | - | - | - | - | - | - | - | - | - | - | - | - | - | - | - | - |
| | **MVM** | *IFA* | ++ | ++ | +++ | +++ | - | - | - | - | - | - | - | - | ++ | + | + | + | - | - |
| | **MPV** | *IFA* | - | - | ++ | +++ | - | - | - | - | - | - | - | - | + | - | - | - | - | - |
| | **MVM** | *HAI* | 1:320 | 1:640 | 1:640 | 1:1280 | | | | | | | | | 1:320 | 1:160 | 1:160 | 1:320 | | |
| | **MPV** | *ELISA* | - | - | - | - | | | | | | | | | - | - | - | - | | |
| | **LCM** | *IFA* | - | - | - | - | | | | | - | - | - | - | - | - | - | - | | |
| | **Adeno (K87)** | *IFA* | - | - | - | - | | | | | - | - | - | - | - | - | - | - | | |
| | **MCMV** | *IFA* | - | - | - | - | - | - | - | - | - | - | - | - | + | + | + | + | - | - |
| | **MCMV** | *ELISA* | | | | | | | | | | | | | pos | pos | - | - | | |
| | **MTV** | *IFA* | - | - | - | - | - | - | - | - | - | - | - | - | - | - | - | - | - | - |
| Bacteria | **Pasteurella pneumotropica** | *IFA* | - | + | + | - | + | - | - | - | - | - | - | - | - | - | + | - | - | - |
| Proto-zoa | **Toxoplasma gondii** | *IFA* | - | - | - | - | - | - | - | - | - | - | - | - | - | - | - | - | - | - |
| | **Encephalitozoon cuniculi** | *IFA* | - | - | - | - | - | - | - | - | - | - | - | - | - | - | - | - | - | - |

[1] BioDoc© Standard Mouse Screening Panel

## B. Parasitological examination of feces[2]

| | FzdM | | | | FzdF | | | | SPF | | | Feral male |
|---|---|---|---|---|---|---|---|---|---|---|---|---|
| Parasites found microscopically | 601 | 606 | 607 | 609 | 801 | 802 | 803 | 810 | 301 | 302 | 309 | 012 |
| Syphacia worms | + | | | + | + | + | | | | | | + |
| Oxyuridae eggs | | | | | | | | | | | | |
| Flagellates | ++ | + | | + | | + | | | | | | ++ |
| Mite eggs | + | | | | | | | | | | | + |

[2] Gross parasitology screening at NMBU, Dept. Food Safety and Infection Biology, Parasitology laboratory
