# Supplementary material for: A Model System for Feralizing Laboratory Mice in Large Farmyard-Like Pens
Source: Front Microbiol. 2021 Jan 11;11:615661. doi: 10.3389/fmicb.2020.615661 (PMC7830425; doi:10.3389/fmicb.2020.615661)
Supplement: Supplementary Figure 1 — Flow cytometry gating strategies. (A) Single cell, mononuclear cells (MNC) and live cell gates. (B) NK cells defined as NKp46+CD3- cells, further defined as maturational stages S1–S4 based on CD27 and CD11b expression, or gated for the expression of KLRG1. (C) T-cells gated equivalent to above, gated as CD4+ or CD8+ and defined as Central Memory (CM; CD62L+CD44+) or Effector Memory (EM; CD62L–CD44+). (D) Regulatory T-cells, gated on CD4+ T-cells equivalent to above, defined as CD25+Foxp3+, and further gated for the expression of Neuropilin-1 (NRP1). (E) In vitro stimulated T-cells, cultured for 48 h in the presence of CD3/CD28 activator beads and IL-2, gated on T-cells equivalent to above and gated for the expression of interferon gamma (IFNg). [file Data_Sheet_1.zip › Supplementary Table S6.pdf]

Supplementary Table S6: Additional serum cytokine measurements

Table shows serum analytes in Exp. 1, where no significant differences were detected between groups, complementary to **Figure 8**.
Values show concentrations (pg/mL). *n*=number of uncensored readings (i.e. within valid range of standard curve).

	**IL-2**		**IL-10**		**IL-17**		**IFN-g**		**TNF-a**		**TGF-b2**		**TGF-b3**	
**Group**	Median (Range)	*n*	Median (Range)	*n*	Median (Range)	*n*	Median (Range)	*n*	Median (Range)	*n*	Median (Range)	*n*	Median (Range)	*n*
SPF	3,8 (2,4-11,3)	*6*	14,0 (5,9-54,1)	*6*	16,9 (2,8-80,13)	*6*	3,3 (2,8-17,7)	*5*	39,0 (15,7-204,5)	*6*	1976,0 (1031,9-2324,9)	*6*	150,7 (70,8-1004,3)	*6*
Fzd^F	2,4 (2,4-6,1)	*3*	65,7 (4,0-678,4)	*5*	5,6 (1,4-491,8)	*5*	6,7 (2,1-8,5)	*5*	136,7 (28,6-3216,2)	*6*	1636,2 (1374,6-6081,9)	*5*	886,5 (111,7-1127,4)	*5*
Fzd^M	5,3 (3,9-23,5)	*5*	60,6 (11,7-98,4)	*6*	8,9 (2,8-296,2)	*6*	6,3 (2,1-72,9)	*6*	93,3 (15,7-1484,2)	*5*	2385,2 (1597,0-2789,4)	*6*	1199,9 189,8-1563,3)	*6*
Feral	6,14	*1*	26,5 (11,2-41,9)	*2*	8,4	*1*	3,6 (1,7-5,6)	*2*	61,1 (53,3-68,8)	*2*	2637,1 (2373,4-2900,9)	*2*	914,1 (754,9-1073,3)	*2*

Analytes falling below lower limit of detection in Exp. 1 and 2:
Exp. 1: IL-1β, IL-4, GM-CSF.
Exp. 2: IL-1β, IL-2, IL-4, IL-9, IL-10, IL-12p70, IL-13, IL-17a, IL-22, IL-23, IL-27, GM-CSF, IFN-γ, TNF-α.
